# Health behavior change in advance care planning: an agent-based model

**DOI:** 10.1186/s12889-016-2872-9

**Published:** 2016-02-29

**Authors:** Natalie C. Ernecoff, Christopher R. Keane, Steven M. Albert

**Affiliations:** Department of Behavioral and Community Health Sciences, University of Pittsburgh, Graduate School of Public Health, 130 DeSoto Street, Pittsburgh, PA 15261 USA; Department of Health Policy and Management, University of North Carolina, Gillings School of Global Public Health, 1101 McGavran-Greenberg Hall, 135 Dauer Drive, Chapel Hill, NC 27510 USA

**Keywords:** Transtheoretical model, Decision making, End-of-life care, Public health, Aging

## Abstract

**Background:**

A practical and ethical challenge in advance care planning research is controlling and intervening on human behavior. Additionally, observing dynamic changes in advance care planning (ACP) behavior proves difficult, though tracking changes over time is important for intervention development. Agent-based modeling (ABM) allows researchers to integrate complex behavioral data about advance care planning behaviors and thought processes into a controlled environment that is more easily alterable and observable. Literature to date has not addressed how best to motivate individuals, increase facilitators and reduce barriers associated with ACP. We aimed to build an ABM that applies the Transtheoretical Model of behavior change to ACP as a health behavior and accurately reflects: 1) the rates at which individuals complete the process, 2) how individuals respond to barriers, facilitators, and behavioral variables, and 3) the interactions between these variables.

**Methods:**

We developed a dynamic ABM of the ACP decision making process based on the stages of change posited by the Transtheoretical Model. We integrated barriers, facilitators, and other behavioral variables that agents encounter as they move through the process.

**Results:**

We successfully incorporated ACP barriers, facilitators, and other behavioral variables into our ABM, forming a plausible representation of ACP behavior and decision-making. The resulting distributions across the stages of change replicated those found in the literature, with approximately half of participants in the action-maintenance stage in both the model and the literature.

**Conclusions:**

Our ABM is a useful method for representing dynamic social and experiential influences on the ACP decision making process. This model suggests structural interventions, e.g. increasing access to ACP materials in primary care clinics, in addition to improved methods of data collection for behavioral studies, e.g. incorporating longitudinal data to capture behavioral dynamics.

**Electronic supplementary material:**

The online version of this article (doi:10.1186/s12889-016-2872-9) contains supplementary material, which is available to authorized users.

## Background

### Advance care planning

Many Americans experience severe illness during which they cannot make health care decisions for themselves [1]. In anticipation of this situation, some individuals opt for advance care planning (ACP), which consists of an individual considering decisions in advance with loved ones and healthcare providers, designating a proxy decision maker, or documenting preferences (or any combination thereof) [2–4]. Preferences for medical intervention vary greatly between patients, and surrogates decision makers are not particularly skilled at making these difficult decisions.

Some research suggests that ACP, specifically speaking with surrogates in advance, may aid in making more patient-centered decisions, and current best practices recommend incorporating surrogates and physicians in the ACP process by discussing patients’ end-of-life values and preferences with them prior to incapacitation [2, 4, 5]. Reasons individuals give for advance care planning include the opportunity to exercise autonomy and control, considering personal relationships, and relieving the burden on loved ones [6]. Surrogates cite a variety of barriers to advance care planning, including the belief that an advance care plan is irrelevant due to perceived health, emotional barriers, relationship concerns, lack of information, and time constraints [7, 8]. This suggests that overcoming the emotional and relational barriers may aid in integrating end-of-life values and preferences into future clinical care [2].

Many patients receiving more intensive treatment than they would want were they able to make their own treatment decisions [9]. Advance directives are associated with more patient-centered end-of-life outcomes [10, 11]. Reducing barriers to ACP and increasing end-of-life planning behaviors may ultimately improve patient-centered end-of-life outcomes. Though interventions exist, ACP rates among older and sicker populations remain relatively low, and understanding the mechanisms of advance care planning may help to target interventions to different stages in the behavior change process. [12].

The primary motivational factors for developing an advance care plan include the diagnosis of oneself or a friend and familiarity with advance care plans and the processes for adopting them [13]. The literature to date has not addressed how best to increase the salience of these motivational factors while reducing barriers associated with ACP.

### The uses of agent-based modeling in advance care planning

Agent-based modeling (ABM), or computational simulations of actions and interactions of autonomous agents (individuals or organizations), allows researchers to examine behavioral dynamics with a computer before implementing interventions for the field [14]. To this end we employ ABM to simulate expected ACP behaviors of individuals and testing interventions on prior to implementing larger-scale public health interventions. ABM methods also allow for the integration of causal dynamics into a simulated population, rather than relying only on analysis of correlations in real populations. The ABM results may aid in justifying time and money allocation to public health programs with the aim of increasing a population’s propensity to develop advance care plans. Though dynamically modeling health behavior change is relatively novel, it has been demonstrated in models of alcohol abuse and child maltreatment, among others [14–18]. ABM has yet to be applied in the context of behavior change in the ACP process.

We developed an ABM depicting ACP as a behavior change process using this Transtheoretical Model framework. Developed by Prochaska and colleagues, the Transtheoretical Model (TTM) has been used widely as a theoretical framework for conceptualizing behavior change, including ACP [8, 19–23]. The TTM places individuals in a stepwise series of readiness states for completing a behavior, where different interventions may be differentially applicable at different stages of readiness. Based on TTM’s conceptual framework, agents move through qualitatively different stages—encountering different barriers and facilitators at each stage—and potentially alter a behavior. We aimed to build an ABM the accurately reflects the rates at which individuals and the population complete the ACP process, barriers (emotional and psychological readiness, having necessary materials), facilitators (increasing salience of the need to develop an ACP, social support), and behavioral variables (susceptibility, baseline distributions).

## Methods

The ABM for ACP contained variables at three levels: individual, social, and structural. Variable and their associated parameters and logic are outlined below and in Table 1. We used NetLogo v5.0.4. for all simulations (Wilensky 1999; [24]). A complete appendix of the NetLogo code can be found in Additional File 1.

**Table 1:** Development of the model

Conceptualization	Logic
• Based on statistics for a population ages 65+ • Baseline ACP behavior distribution (from literature) o % pre-contemplation o % contemplation o % preparation o % action-maintenance • Cut-points (on 0–100 scale) determine each of TTM stages o Each stage consists of a different (not equally-distributed) point range o Based on different difficulties to move up in TTM stage • Agents move each day	Distributed to fit percentages (0–100) based on TTM Sliders for each of 5 stages to determine starting distribution ACP propensity based on a changing number of points (0–100 scale) per individual; varying cut points to designate Threshold rules for moving up stages Turtle changes color at action stage Each tick equals 1 day Move for at least 5 years
Dynamic Modeling of Experiences	Logic
• Personal critical illness o Smaller patch (less likely) o Higher impact factor (one’s own severe illness likely has a greater impact on Death Planning Anxiety) • Loved one’s critical illness/death o Larger patch (more likely to know someone who has had severe illness) o Smaller impact factor (the experiences of others likely have a lesser impact on Death Planning Anxiety) • Advance care planning discussion with primary care provider o Relative small influence, based on non-urgency of the primary care setting	1 patch for each event (personal illness, loved one’s illness, and primary care interaction) Sliders to indicate degree of impact for each Probability of affecting ACP change when land on patches can vary (sliders 0–100 indicate likelihood) • If gain points, then count points • If count > next TTM threshold, then move to higher stage • If count < next TTM threshold, then stay in current stage If move up stage, then reevaluate current stage • If in Action-Maintenance stage, then turn designated color • If not in Action-Maintenance stage, then retain color
Dynamic Modeling of Social Interactions	Logic
• Interactions with other individuals • Recognize level of ACP • Susceptibility (not all agents are impacted by other agents) • At each tick, evaluate any agents on same patch • At each tick, if patch-mate in higher stage, then gain interaction points o If neighbors, then evaluate for higher stage than self o If neighbor at high stage, then probability of assign associated number of points o Susceptibility: slider-based probability at agent level o Each stage associated with a number of points gained by lower stages upon interaction • Local Networks o Observable connections between agents that interact o Agents move at a constant rate, from patch to patch in random directions (in contrast to randomly across entire matrix) • Backsliding (negative social interaction) o Negative social influence can accumulate o With a sufficient accumulation of negative points, agents can cross the threshold back into the previous stage	If interact with neighbor, increase ACP propensity for lesser neighbor Different degrees of disparity will have a different levels of influence If gain points, then count points • If count > next TTM threshold, then move to higher stage • If count < next TTM threshold, then stay in current stage If on same patch, then make connection with agent At each tick, move at random 360° and move forward at designated moving-rate If move up stage, then reevaluate current stage • If in Action-Maintenance stage, then turn designated color • If not in Action-Maintenance stage, then retain color • If interact with neighbor, decrease ACP propensity for higher neighbor
Susceptibility	Logic
• Not all agents are impacted by experiences and social interactions	• If land on patch, then probability of gaining points

### Overview of the dynamic agent-based model

We built an ABM depicting individuals who progress through the Transtheoretical Model’s (TTM) stages of change. Our simulated population begins with a distribution of stages of change for ACP empirically found in other behaviors. Within each stage, agents have a score (with a possible range of 0–100), with higher scores representing greater likelihood of advancing to the next successive stage of change.

We endowed our simulated population with relevant variables found in prior literature (Table 2) [19, 21]. The simulated individuals can experience events (described below). At each time step, each day, every simulated individual probabilistically encounter (1) other individuals and (2) life events. Wherever possible, we determined the probability of life events from the literature. These probabilistic encounters with other agents and with life events then determined (non-probabilistic) the agent’s propensity score. When a pre-specified threshold of a propensity scores is reached, the individual deterministically moves to the next or previous stage (Table 2)

**Table 2:** Variables in the model

Baseline distribution of agents across stages	Bounds	SIM 1	SIM 2	Expected^a^
pre-contemplation	0–100	100	40^b^	
contemplation	0–100	0	40^b^	
preparation	0–100	0	20^b^	
action-maintenance	0–100	0	0^b^	
Baseline point value for each stage				
pre-contemplation	0–100	0	100	
contemplation	0–100	0	50	
preparation	0–100	0	0	
action-maintenance	0–100	0	50	
Thresholds				
contemplation	0–100	60	100	
preparation	0–100	20	50	
action-maintenance	0–100	10	0	
Points				
Experiences ICU stay	0–10	4	6	
Experiences loved one’s illness	0–10	3	4	
Interacts with other agents at higher stages	0–10	3	2	
Interacts with other agents at lower stages	−10–0	1	2	
Visits primary care (PCP)	0–10		1	
Globals				
Probability of experiencing ICU stay	0–10	3	3	
Probability of experiencing loved one’s illness	0–10	6	7	
Probability of interacting with primary care	0–10		10	
Other Parameters				
Agents in pre-contemplation are not influenced by other agents’ stages upon interaction				
Susceptibility	0–100	100	50	
Movement rate in local networks			0.15	
Outcomes				
%pre-contemplation	0–100	0	21.4	40
%contemplation	0–100	0	20.4	10
%preparation	0–100	0	6.8	3
%action-maintenance	0–100	100	51.4	47

^a^based on [21] ^b^based on [19]

#### Pre-contemplation

Agents in the pre-contemplation stage have never considered ACP. In the general population, these are people who have never been introduced to the ACP process, are not aware of related concepts, or have been introduced, but do not find ACP to be a worthwhile or relevant behavior. Agents in the pre-contemplation stage are not engaged in ACP in any respects.

#### Contemplation

Agents in the contemplation stage begin to think about their treatment preferences and values. They are not yet ready to talk about their thoughts or take action with respect to planning behaviors. Barriers to entering this stage from pre-contemplation include the perceived irrelevance of ACP for various reasons, including the idea that one is too healthy. An additional barrier is the desire to leave determinations of life and death in “God’s hands.”

#### Preparation

The preparation stage consists of those who have decided ACP would be an advantageous behavior for them. These people begin clarifying their values by talking to healthcare providers and loved ones. They develop a plan to formally discuss end-of-life decisions with their surrogate decision makers and healthcare team. Barriers to preparation include a lack of resources or education about what is required in the ACP process. Additionally, emotional and psychological barriers influence one’s willingness to discuss these issues and prepare for end-of-life scenarios. As in contemplation, if individuals perceive themselves as too healthy, they may rank follow-through with the ACP behavior below other aspects of their lives, citing that they are too busy.

#### Action-maintenance

The final stage is action, or completion of the health behavior. Those agents in action-maintenance completed the initial behavior and continue to maintain and update their ACP. Once the ACP behavior is completed, agents enter the action-maintenance stage. If they fail to maintain it (i.e. not updating annually), they relapse, out of action-maintenance, into preparation.

Agents in the action-maintenance stage have had active discussion with their family and physician. This discussion can be documented in the form of an advance directive. Wishes are then reviewed annually and amended as necessary. Barriers to entering the action-maintenance stage include the inaccessibility or unwillingness of loved ones or healthcare providers to discuss end-of-life wishes. Likewise, some do not have potential surrogate decision makers. Emotional and psychological barriers at this stage also include the desire to not burden loved ones with such a discussion. With respect to maintaining active status, some individuals disregard or are not aware of the need to review and update advance care plans.

### Dynamic modeling of experiences

Based on the Transtheoretical Model and evidence from the literature, we incorporated key barriers and facilitators into the ABM that may influence perceptions. Specifically, agents could survive a stay in an intensive care unit (ICU) with high probability of death or severe functional impairment, or experience the death of a loved one. In the model, experiences were represented by patches that agents randomly move to (described below). Agent experience of these life events potentially causes them to earn ACP propensity points, advancing them forward or backward to a neighboring stage of change.

#### Exposure to personal critical illness

We simulated effects of critical illness experience on perceived health. Previous literature has noted one of the major barriers to ACP is that people often perceive themselves as too healthy to need an ACP [8]. The personal critical illness occurred relatively infrequently in the overall population, as the average person over 65 years of age is likely to not experience an intensive care unit stay very frequently. Though infrequent, when these events occur, they have a relatively high influence on one’s development of an ACP.

In the model, the probability for personal illness is relatively small (in comparison to the probability of the critical illness or death of a loved one, outlined below), given their relative infrequency. It also carry a larger weight, meaning susceptible agents who land on the personal illness patches gain relatively more ACP points given its presumably greater influence on future behavior with respect to ACP.

#### Exposure to a loved one's critical illness

We also included the influence of a loved one’s severe illness or death. Similar to personal experience, this encounter with illness or death is intended to address the barrier of applicability. Barriers presented by Schickedanz and colleagues include both perceived health (as noted above) and the perception that one is too busy to complete an ACP [8]. Both of these concepts can be addressed by reexamination and reprioritization. If perceived necessity increases based on life events, a person may be more likely to develop an ACP. Therefore, in the ABM a loved one’s critical illness or death is more probable than having a personal encounter with critical illness (i.e. individuals are only one person and they know more than one person, making the latter more probable). These secondhand encounters have less impact on propensity to develop an ACP by virtue of one’s proximity to the situation in personal experience and the salience that comes with such an event.

#### Exposure to a primary care provider

We included a primary care influence, as that is the forum in which most advance care planning discussions occur with providers. These encounters are likely the least influential in prompting discussion of end-of-life preferences, as the sense of urgency is lessened, making the behavior seem less applicable to the current setting [8]. Although advance care planning is likely not discussed in each encounter with primary care, individuals over 65 encounter providers in this setting more frequently than they do in the ICU setting [25], so the probability of this encounter is the largest of the experiences modeled in the ABM. The fact that advance care planning is not discussed in each encounter is captured by the low influence the patch has on agents in the model per encounter.

The primary care patch is also placed relatively nearer to the ICU patch than that representing the death or critical illness of a loved one. We intentionally placed configured the placement in this way to reflect that patients who were critically ill are more likely to seek out primary care than when a loved one has a similar experience. Patients are often referred to primary care or other outpatient clinical care for follow-up after critical illness, during which providers are more likely to address ACP given the patient’s previous exposure.

### Dynamic modeling of social interactions

If an agent moves to a patch with another individual they “talk” to this other individual about ACP. Talking to another individual may bias the agent for or against ACP. Individuals who occupy higher stages (presumably in favor of ACP as a concept) are able to influence those in lower stages, making them more likely to complete an ACP. Likewise, those at relatively lower stages negatively influence those of higher stages. The influence of those in lower stages can result in agents returning to previous stages.

After a primary analysis in which agents moved in random networks, the model was expanded to allow agents to build local networks and move within a relatively more structured community. In the model, local networks were implemented by requiring agents to move one geographic space at a time (i.e. take one step up, down, left, right, or diagonally) in a random fashion to maintained a more constant environment with respect to the other agents surrounding it.

### Susceptibility

We built susceptibility into the model to demonstrate observations that some individuals in the population will not complete an advance care plan regardless of experiences or interactions. A subset of the agents will not be affected by the influencing factors in the model.

### Experiments

We ran two types of experiments systematically to simulate different dynamics: (1) a model in which all agents developed an advance care plan and (2) models in which the agents developed advance care plans under the influence of each other, ICU stay, and loved ones’ illness or death, visits to primary care clinics and the addition of local networks. The first experiment was designed to represent how transitions between stages would occur in an ideal situation. The second experiment was designed to mimic observed population rates of ACP in the TTM.

For each of the experiments, we systematically manipulated five sets of variables in the model: initial distribution across stages, initial scores within each stage, percent susceptible, points for experiences and interactions, and score thresholds to move between stages.

In each experiment, we varied the parameters to determine the weight with which they may influence agents. Baseline values were determined based upon values found in the literature when available, the results for which are presented here.

## Results

Upon evaluating the resulting distributions across the stages of change, we were able to determine the sets of parameters that best match those found at the population level for each of the four simulations (Table 2).

### Simulation 1

**Fig. 1:**
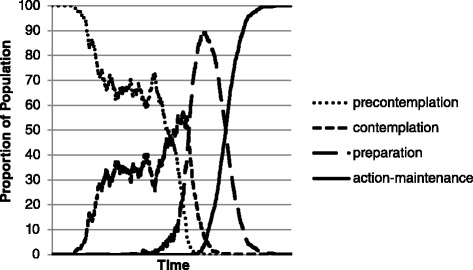
Results from Simulation 1: All agents start in precontemplation and move through all of the stages of change to action-maintenance

The first simulation aimed to represent the means by which our ABM can appropriately reflect the progress of a population of individuals who all complete the ACP process. A graph of the transition rates can be found in Fig. 1.

**Fig. 2:**
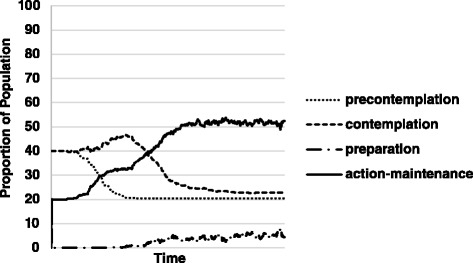
Results from Simulation 2: Agents start at a baseline distribution common in generalized health behavior change. Agents approach the distribution across the stages of change found in data specific to ACP

As a baseline for comparison, all agents started in pre-contemplation, and all agents in the model were susceptible to experiences that could change their behavior. We found all agents progressed from pre-contemplation to action-maintenance and remained there. Agents wavered between pre-contemplation and contemplation before progressing into preparation and subsequently moving rather quickly from preparation to action-maintenance. The ABM plausibly shows progression through stages at the individual level.

Starting in pre-contemplation, some agents are affected by interactions and life events relatively early and start to move into contemplation. As the number of agents in contemplation increases, interactions between the first two stages become more relevant, producing stochastic interactions between the two, indicating high interaction influence early in the behavioral process. Agents move through preparation relatively quickly, as the threshold to enter it is high and the threshold to move out of preparation is low, based on the nature of the barriers and facilitators for the stage found in the literature. Once preparation begins, backsliding due interactions with those in pre-contemplation becomes less likely. This is likely due to a relatively low threshold in ACP to complete the behavior (action-maintenance; red) once a decision has been made to do so. That is, once an agent decides to complete the ACP behavior during contemplation, that agent does not have to expend much effort to prepare and move quickly to action-maintenance.

We were able to accurately reflect the relative rates at which individuals move through the stages of change when all complete the process. In this experiment, all agents were susceptible to the events and interactions, meaning they all progressed to action-maintenance. Additionally, all agents started in pre-contemplation. These two factors in conjunction forced all agents through all four of the stages in the model. The rate and pattern with which agents made the transition offers one illustration of the barriers and facilitators associated with ACP and how certain experiences and social interactions may alter individuals’ progression through the TTM’s stages of change.

### Simulation 2

Starting with a distribution typically found in the general population for multiple health behaviors, agents gradually redistribute across the stages of change. The simulation incorporated social influence in local networks, ICU stay, and loved ones’ illness or death, and visits to primary care clinics. Agents’ progression through stages can be found in Fig. 2.

We were able to accurately reflect the relative rates and more accurately reflect the distribution within the stages of change at which individuals complete the ACP process relative to population values [21]. The observed and expected values for each stage relative to the general population can be found in Table 2.

This second type of simulation shows that our ABM can reflect ACP practices in the population. Integrating local networking allowed the model to potentially represent dynamics more similarly to those found in the population. Agents achieved a distribution among the stages of change relatively representative of population values

## Discussion

Based upon the current literature in ACP that utilizes the Transtheoretical Model in human populations, our model provides one plausible representation of how individuals may make decisions to complete an advance care plan. Schickedanz et al. conducted a qualitative study to identify key barriers to advance care planning among older adults, and the key barriers they identified were incorporated into the model [8]. The same group administered a survey to a similar population to determine how people who could reasonably complete an advance care plan were distributed across the Transtheoretical Model’s stages of change [21]. The combination of data from these two studies shaped how we conceived barriers, facilitators, and readiness for the model.

In the ABM individual agents moved through the four modified stages of the Transtheoretical Model as one would expect based on previous use of the stages of change model in population settings (Fig. 2).

Starting in pre-contemplation, some agents experience the life events relatively early and start to move into contemplation. As the number of agents in contemplation increases, interactions between the first two stages become more relevant, producing stochasticity between the two as perceived emotional barriers and social norms fluctuate. Eventually, contemplation is able to overcome pre-contemplation as some of its constituents reach the threshold for the third stage, preparation.

As preparation is a relatively fast stage to move through once it has been entered, backsliding to an earlier stage, due to interactions with those in pre-contemplation becomes less likely. This is likely due to a relatively low effort in ACP to complete the behavior (action-maintenance) once a decision has been made to more forward (represented by presence in preparation), that is, once an agent decides to complete the ACP behavior that agent does not have to expend much effort to prepare and move quickly to action-maintenance. Some individuals relapse from the action stage by not annually updating the ACP.

As individuals are able to move through the preparatory stage quickly, action maintenance increases as subjects create ACPs.

### Strengths of the model

The ACP model offers a generalizable method for integrating the Transtheoretical Model into an ABM. The novel application of ABM can be adapted to other health behaviors by adjusting the barriers and facilitators affecting movement through the stages of change.

Dynamic ABM facilitates the presentation of potentially causal pathways for a behavior. We were able to find a sufficient mechanism in the model for recreating at least some of the empirical results. Though it is unknown if this mechanism drives ACP behavior, the model’s strength lies in its ability to integrate, vary, and test potentially causal factors of a behavior, giving it high internal validity.

The behavior change model has high internal validity by definition because agents behave according to model assumptions. By altering parameters using values ascertained from the literature, we developed a best-fit model to predict ACP population estimates.

Models offer the benefit of highlighting gaps in the literature where more research is necessary. Our model provides one potential mechanism for advance care planning, and additional empirical research can act to strengthen the model.

### Limitations of the model

Our model offers one potential, sufficient mechanism for achieving the ACP rates found empirically. There are likely other mechanisms sufficient to produce the same results. We acquired estimates from the literature for some variables: target distribution across stages of change, the most impactful factors for prompting ACP (death of a family member or friend, hospital stay, prompt by primary care), and the importance of interaction with others; many variables were not currently available in literature, and additional empirical research can inform future iterations of the model. We only included three of many factors that may influence propensity to develop an advance care plan. Our agents do not have families or individualized networks, nor do they have demographic or other sociologic characteristics such as age, socio-economic status, race, religion, chronic health status, and attitudes toward medicine. The effects of all of these variables can be better described empirically and integrated into the ABM to provide a clearer picture of health behavior change. The integration of additional barriers and facilitators of ACP may help the model better reflect population-level decision making.

### Suggestions for future studies

To the authors’ knowledge, studies on advance care planning behavior have been cross sectional, capturing one time point in the process. Future studies can be longitudinal in nature, capturing individual behavior across a time period. Such studies will lend insight with respect to the advance care planning process and individuals’ dynamic movement through it. Detailed data on the influences and execution of behaviors, including detailed local network structures, can aid in the development of interventions designed to prompt advance care planning behavior in the population.

Longitudinal data can inform investigators of potential intervention points to introduce effective facilitators or intervene on existing barriers. Such interventions may include increasing the prevalence with which ACP is discussed in primary or specialty care clinics, providing resources in skilled nursing facilities or community centers. Likewise, decisional support tools and workshops may prompt individuals and their families to learn about ACP and complete the behavior.

## Conclusion

Our ABM is a useful method for representing dynamic social and experiential influences on the ACP decision making process. This model suggests structural interventions, e.g. increasing access to ACP materials in primary care clinics, in addition to improved methods of data collection for behavioral studies, e.g. incorporating longitudinal data to capture behavioral dynamics. ABM may allow for the testing of ACP interventions prior to implementation as a way of testing for effectiveness prior to allocation decisions.

## Additional file

Additional file 1:
**Netlogo Code for Health Behavior Change in Advance Care Planning.** (DOCX 21 kb)

## Abbreviations

ABM: agent-based model
ACP: advance care planning
