# Supplementary material for: Health behavior change in advance care planning: an agent-based model
Source: BMC Public Health. 2016 Feb 29;16:193. doi: 10.1186/s12889-016-2872-9 (PMC4770523; doi:10.1186/s12889-016-2872-9)
Supplement: Additional file 1: — Netlogo Code for Health Behavior Change in Advance Care Planning. (DOCX 21 kb) [file 12889_2016_2872_MOESM1_ESM.docx]

**Additional file 1**

Netlogo Code for Health Behavior Change in Advance Care Planning

globals [

primary-affected-patches

secondary-affected-patches

PCP-patches

%precontemplation

%contemplation

%preparation

%action-maintenance

total-action-maintenance

%primary-affected

%secondary-affected

%PCP

]

turtles-own [

ACP-proclivity-count

duration

contemplation-score

precontemplation-score

preparation-score

action-maintenance-score

precontemplation?

contemplation?

preparation?

action-maintenance?

susceptible? ; either yes or no or change this to be a continuous variable e.g. 0-100 then probabilistic

]

patches-own [

primary-affected-here?

secondary-affected-here?

PCP-here?

]

to setup

clear-all

setup-patches

create-turtles NumberTurtles

setup-turtles

reset-ticks

end

to setup-turtles

ask turtles [setxy random-xcor random-ycor]

set-default-shape turtles "person"

ask turtles [ifelse random 100 < %susceptible [set susceptible? true] [set susceptible? false]]

;; initialize all as false first.

ask turtles [

set precontemplation? false set contemplation? false set preparation? false set action-maintenance? false

set color white ;; white means no stages. Sliders should add to 100%.

set precontemplation-score initial-precontemplation-score

set contemplation-score initial-contemplation-score

set preparation-score initial-preparation-score ;; could change this to zero for all, but more flexible to set some initial value

set action-maintenance-score initial-action-maintenance-score

]

ask turtles [if who < initial%precontemplation * count turtles / 100

[set precontemplation? true set color yellow ]]

ask turtles [if who >= initial%precontemplation * count turtles / 100 and who < (initial%contemplation * count turtles / 100 ) + (initial%precontemplation * count turtles / 100 )

[set contemplation? true set color green ] ]

ask turtles [if who >= (initial%contemplation * count turtles / 100) + (initial%precontemplation * count turtles / 100 )

and who < (initial%preparation * count turtles / 100 ) + (initial%precontemplation * count turtles / 100 ) + (initial%contemplation * count turtles / 100)

[set preparation? true set color blue]]

ask turtles [if who >= (initial%preparation * count turtles / 100) + (initial%contemplation * count turtles / 100) + (initial%precontemplation * count turtles / 100 )

and who < (initial%action-maintenance * count turtles / 100 ) + (initial%preparation * count turtles / 100 ) + (initial%precontemplation * count turtles / 100 ) + (initial%contemplation * count turtles / 100)

[set action-maintenance? true set color red]]

;; Sliders must add to 100%

;; update globals here as well as in 'go' to see proportions in each stage displayed in four monitors.

update-global-variables

update-colors

end

to setup-patches

clear-all

ask patches [set primary-affected-here? false set secondary-affected-here? false set PCP-here? false

ifelse pxcor < 0 [set pcolor black] [set pcolor black]]

set primary-affected-patches patches with

[pxcor > (16 - primary-affected-patch) and pycor < (-16 + primary-affected-patch)]

ask primary-affected-patches [set primary-affected-here? true set pcolor pink]

set secondary-affected-patches patches with

[pxcor > (16 - secondary-affected-patch) and pycor > (16 - secondary-affected-patch)]

ask secondary-affected-patches [set secondary-affected-here? true set pcolor orange]

set PCP-patches patches with

[pxcor < (-16 + PCP-patch) and pycor > (16 - PCP-patch)]

ask PCP-patches [set PCP-here? true set pcolor violet]

end

to update-global-variables

if count turtles > 0

[set %precontemplation (count turtles with [precontemplation? = true] / count turtles * 100)

set %contemplation (count turtles with [contemplation? = true] / count turtles * 100)

set %preparation (count turtles with [preparation? = true] / count turtles * 100)

set %action-maintenance (count turtles with [action-maintenance? = true] / count turtles * 100)

]

end

to affect-score-turtles

ask turtles with [susceptible?] [if [primary-affected-here?] of patch-here and precontemplation? = true [set contemplation-score contemplation-score + primary-points]]

ask turtles with [susceptible?] [if [primary-affected-here?] of patch-here and contemplation? = true [set preparation-score preparation-score + primary-points]]

ask turtles with [susceptible?] [if [primary-affected-here?] of patch-here and preparation? = true [set action-maintenance-score action-maintenance-score + primary-points]]

ask turtles with [susceptible?] [if [primary-affected-here?] of patch-here and action-maintenance? = true and action-maintenance-score < 100 [set action-maintenance-score action-maintenance-score + primary-points]]

ask turtles with [susceptible?] [if [secondary-affected-here?] of patch-here and precontemplation? = true [set contemplation-score contemplation-score + secondary-points]]

ask turtles with [susceptible?] [if [secondary-affected-here?] of patch-here and contemplation? = true [set preparation-score preparation-score + secondary-points]]

ask turtles with [susceptible?] [if [secondary-affected-here?] of patch-here and preparation? = true [set action-maintenance-score action-maintenance-score + secondary-points]]

ask turtles with [susceptible?] [if [secondary-affected-here?] of patch-here and action-maintenance? = true and action-maintenance-score < 100 [set action-maintenance-score action-maintenance-score + secondary-points]]

ask turtles with [susceptible?] [if [PCP-here?] of patch-here and precontemplation? = true [set contemplation-score contemplation-score + PCP-points]]

ask turtles with [susceptible?] [if [PCP-here?] of patch-here and contemplation? = true [set preparation-score preparation-score + PCP-points]]

ask turtles with [susceptible?] [if [PCP-here?] of patch-here and preparation? = true [set action-maintenance-score action-maintenance-score + PCP-points]]

ask turtles with [susceptible?] [if [PCP-here?] of patch-here and action-maintenance? = true and action-maintenance-score < 100 [set action-maintenance-score action-maintenance-score + PCP-points]]

end

to interact

ask turtles with [susceptible?] [if contemplation? and any? turtles-here with [preparation? = true] [set contemplation-score contemplation-score + interact-points]]

ask turtles with [susceptible?] [if contemplation? and any? turtles-here with [action-maintenance? = true] [set contemplation-score contemplation-score + interact-points]]

ask turtles with [susceptible?] [if preparation? and any? turtles-here with [action-maintenance? = true] [set preparation-score preparation-score + interact-points]]

ask turtles with [susceptible?] [if preparation? and any? turtles-here with [preparation? = true] [set preparation-score preparation-score + interact-points]]

;;ask turtles with [susceptible?] [if action-maintenance? and any? turtles-here with [action-maintenance? = true] [set action-maintenance-score action-maintenance-score + interact-points]]

ask turtles with [susceptible?] [if contemplation? and any? turtles-here with [precontemplation? = true] [set contemplation-score contemplation-score - negative-interact-points]]

ask turtles with [susceptible?] [if preparation? and any? turtles-here with [precontemplation? = true] [set preparation-score preparation-score - negative-interact-points]]

ask turtles with [susceptible?] [if action-maintenance? and any? turtles-here with [precontemplation? = true] [set action-maintenance-score action-maintenance-score - negative-interact-points]]

ask turtles with [susceptible?] [if action-maintenance? and any? turtles-here with [contemplation? = true] [set action-maintenance-score action-maintenance-score - negative-interact-points]]

end

to ACP-affect-score-turtles ;;move turtles up in stage based on stage-specific point accumulation; theshold determined by stage-specific sliders

ask turtles [if precontemplation? and contemplation-score > score-threshold-contemplation - 20

[

set contemplation? true

set precontemplation? false

set preparation? false

set action-maintenance? false

]]

ask turtles [if contemplation? and preparation-score > score-threshold-preparation - 20

[

set preparation? true

set precontemplation? false

set contemplation? false

set action-maintenance? false

]]

ask turtles [if preparation? and action-maintenance-score > score-threshold-action-maintenance - 20

[

set action-maintenance? true

set precontemplation? false

set preparation? false

set contemplation? false

]]

ask turtles [if contemplation-score < 0 [set contemplation-score 0]]

ask turtles [if preparation-score < 0 [set preparation-score 0]]

ask turtles [if action-maintenance-score < 0 [set action-maintenance-score 0]]

ask turtles [if action-maintenance? and action-maintenance-score <= 0

[

set precontemplation? false

set contemplation? false

set preparation? true

set action-maintenance? false

]]

ask turtles [if preparation? and preparation-score <= 0

[

set precontemplation? false

set contemplation? true

set preparation? false

set action-maintenance? false

]]

ask turtles [if contemplation? and contemplation-score <= 0

[

set precontemplation? true

set contemplation? false

set preparation? false

set action-maintenance? false

]]

end

to affect-ACP-score

ask turtles with [susceptible?] [if [primary-affected-here?] of patch-here and precontemplation? = true [set contemplation-score contemplation-score + primary-points]] ; if random 100 < 50 [

ask turtles with [susceptible?] [if [primary-affected-here?] of patch-here and contemplation? = true [set preparation-score preparation-score + primary-points]]

ask turtles with [susceptible?] [if [primary-affected-here?] of patch-here and preparation? = true [set action-maintenance-score action-maintenance-score + primary-points]]

ask turtles with [susceptible?] [if [secondary-affected-here?] of patch-here and precontemplation? = true [set contemplation-score contemplation-score + secondary-points]]

ask turtles with [susceptible?] [if [secondary-affected-here?] of patch-here and contemplation? = true [set preparation-score preparation-score + secondary-points]]

ask turtles with [susceptible?] [if [secondary-affected-here?] of patch-here and preparation? = true [set action-maintenance-score action-maintenance-score + secondary-points]]

ask turtles with [susceptible?] [if [PCP-here?] of patch-here and precontemplation? = true [set contemplation-score contemplation-score + PCP-points]]

ask turtles with [susceptible?] [if [PCP-here?] of patch-here and contemplation? = true [set preparation-score preparation-score + PCP-points]]

ask turtles with [susceptible?] [if [PCP-here?] of patch-here and preparation? = true [set action-maintenance-score action-maintenance-score + PCP-points]]

end

to move-turtles

;;ask turtles [move-to one-of patches]

if encounters = "correlated"

[ rt random 360

fd moving-rate ]

if encounters = "random" [move-to one-of patches]

if link-up [link-up-turtles]

end

to link-up-turtles

if any? other turtles-here [create-links-with other turtles-here]

end

to update-colors

ask turtles [if precontemplation? = true [set color yellow]]

ask turtles [if contemplation? = true [set color green]]

ask turtles [if preparation? = true [set color blue]]

ask turtles [if action-maintenance? = true [set color red]]

end

to go

ask turtles

[move-turtles]

affect-score-turtles

ACP-affect-score-turtles

interact

update-colors

update-global-variables

tick

end

;;setting totals

;to update-globals

; set %contemplation (count turtles with [contemplation? = true]) / count turtles

; set %precontemplation (count turtles with [precontemplation? = true]) / count turtles

;end
